# Cytotoxicity, Uptake Behaviors, and Oral Absorption of Food Grade Calcium Carbonate Nanomaterials

**DOI:** 10.3390/nano5041938

**Published:** 2015-11-10

**Authors:** Mi-Kyung Kim, Jeong-A. Lee, Mi-Rae Jo, Min-Kyu Kim, Hyoung-Mi Kim, Jae-Min Oh, Nam Woong Song, Soo-Jin Choi

**Affiliations:** 1 Department of Food Science and Technology, Seoul Women’s University, 621 Hwarang-ro, Nowon-gu, Seoul 139-774, Korea; E-Mails: mermaidp-mk@hanmail.net (M.-K.K.); junga0462@hanmail.net (J.-A.L.); mirae8651@naver.com (M.-R.J.); 2 Department of Chemistry and Medical Chemistry, College of Science and Technology, 1 Yonseidaegil, Wonju, Gangwondo 220-710, Korea; E-Mails: ipz9rv@naver.com (M.-K.K.); annabb@hanmail.net (H.-M.K.); jaemin.oh@yonsei.ac.kr (J.-M.O.); 3 Center for Nanosafety Metrology, Korea Research Institute of Standards and Science, 267 Gajeong-ro, Yuseong-gu, Daejeon 305-340, Korea; E-Mail: nwsong@kriss.re.kr

**Keywords:** calcium carbonate, cytotoxicity, cellular uptake, intestinal transport, oral absorption

## Abstract

Calcium is the most abundant mineral in human body and essential for the formation and maintenance of bones and teeth as well as diverse cellular functions. Calcium carbonate (CaCO_3_) is widely used as a dietary supplement; however, oral absorption efficiency of CaCO_3_ is extremely low, which may be overcome by applying nano-sized materials. In this study, we evaluated the efficacy of food grade nano CaCO_3_ in comparison with that of bulk- or reagent grade nano CaCO_3_ in terms of cytotoxicity, cellular uptake, intestinal transport, and oral absorption. Cytotoxicity results demonstrated that nano-sized CaCO_3_ particles were slightly more toxic than bulk materials in terms of oxidative stress and membrane damage. Cellular uptake behaviors of CaCO_3_ nanoparticles were different from bulk CaCO_3_ or Ca^2+^ ions in human intestinal epithelial cells, showing efficient cellular internalization and elevated intracellular Ca^2+^ levels. Meanwhile, CaCO_3_ nanoparticles were efficiently transported by microfold (M) cells *in vitro* model of human intestinal follicle-associated epithelium, in a similar manner as Ca^2+^ ions did. Biokinetic study revealed that the biological fate of CaCO_3_ particles was different from Ca^2+^ ions; however, *in vivo*, its oral absorption was not significantly affected by particle size. These findings provide crucial information to understand and predict potential toxicity and oral absorption efficiency of food grade nanoparticles.

## 1. Introduction

Calcium is essential for the formation and maintenance of bones and teeth, cellular physiology, immune response, hormone secretion, activation of enzymes, and blood-clotting system [1,2]. Calcium carbonate (CaCO_3_) is the most prevalent form of a calcium supplement due to its abundance from nature, such as oyster and sea shells, as well as the most cost-effective [3]. However, oral bioavailability of CaCO_3_ is extremely low, since calcium is well absorbed into the body under acidic conditions and an alkaline CaCO_3_ requires stomach acid for better absorption [3,4,5]. Moreover, solubility of CaCO_3_ is known to be generally low compared to other inorganic nanoparticles [6]. The low efficacy of CaCO_3_ may be overcome by applying nano-sized materials, which can induce cellular uptake by endocytosis [7,8], different uptake pathways from that for Ca^2+^ ions [9]. Much research has demonstrated that nanoparticles can be internalized into cells by energy-dependent endocytosis, consequently contributing to enhanced uptake efficacy [10,11,12].

In order to obtain food grade CaCO_3_ particles, a mechanical grinding process from oyster or sea shells, namely the top-down approach, is generally applied. However, along with extensive application of nanotechnology to diverse fields, increasing concern about potential adverse effects of nanomaterials on human body has been raised, needing verification on their toxicity [13,14]. According to The United States Toxic Substances Control Act inventory, a lethal dose of 50% (LD_50_) of CaCO_3_ is 6450 mg/kg), being classified in the group with the least toxicity. Indeed, Jeong *et al.* demonstrated that nano CaCO_3_ did not cause toxicity up to 2000 mg/kg after a 14-day oral repeated dose administration to rats, supporting its low toxicity [15]. Moreover, biological fates of nanoparticles are necessary to be determined, especially for nanomaterials that can be partially dissolved into ions under physiological condition, in order to understand and predict their potential toxicity and toxic mechanisms [16,17,18]. On the other hand, conflicting results have been reported on increased bioavailability of CaCO_3_ nanoparticles compared to that of bulk materials [19,20,21], and, therefore, further elucidation on enhanced efficacy of nanoparticles through the gastrointestinal tract is necessary [22,23].

In this study, we investigated the effects of particle size (bulk *versus* nano) of food grade CaCO_3_ particles (food bulk CaCO_3_ and food nano CaCO_3_), both produced by grinding sea shells, on cytotoxicity, cellular uptake behaviors, and intestinal transport. Furthermore, oral bioavailability of CaCO_3_ particles with respect to particle size was evaluated after a single-dose oral administration to rats. A comparative study with reagent grade CaCO_3_ particles (SS CaCO_3_), which was produced by the bottom-up approach of high gravity reactive precipitation, or Ca^2+^ ions was also performed to compare biological responses of CaCO_3_ particles prepared by different methods and to answer the question as to whether the effects of CaCO_3_ particles result from particulate forms or ionic forms in the biological system.

## 2. Results and Discussion

### 2.1. Characterization

**Figure 1:**
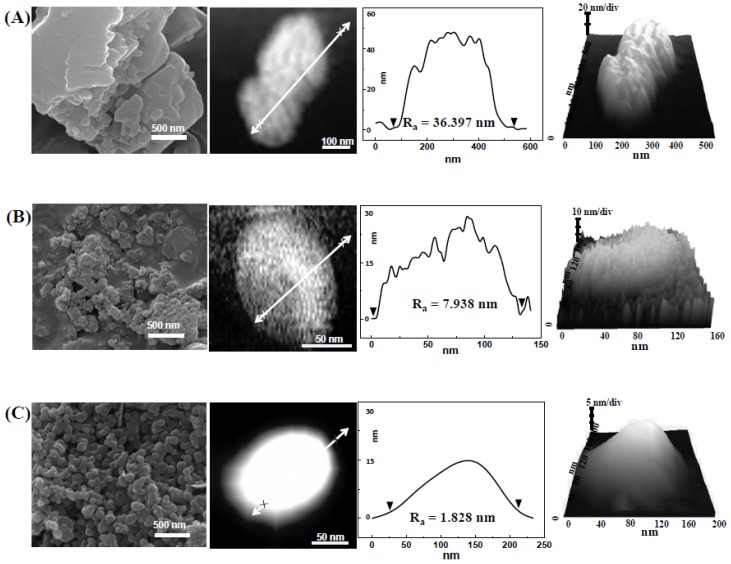
Scanning electron microscopy (SEM) images, atomic force microscope (AFM) images, height profiles, and 3D images of (**A**) food bulk CaCO_3_; (**B**) food nano CaCO_3_; and (**C**) SS CaCO_3_.

Figure 1 demonstrates scanning electron microscopy (SEM) images of three different CaCO_3_ particles. Particle size of food bulk CaCO_3_ particles was heterogenous, showing an irregular shape, while food nano CaCO_3_ and reagent grade SS CaCO_3_ had relatively homogenous size distributions. An average primary particle size of food bulk CaCO_3_, food nano CaCO_3_, and SS CaCO_3_ were determined to be ~2 μm, ~100 nm, and ~110 nm, respectively. Specific surface areas measured by nitrogen adsorption-desorption isotherm were 1, 16 and 21 m^2^/g for food bulk CaCO_3_, food nano CaCO_3_, and SS CaCO_3_, respectively, indicating that smaller particles tend to have larger specific surface area. Slightly larger surface area of SS CaCO_3_ than food nano CaCO_3_ in spite of larger primary particle size of the former rather than the latter, was thought to be originated from the uniform particle size distribution of SS CaCO_3_. On the other hand, SEM images showed that food bulk CaCO_3_ and food nano CaCO_3_ had different surface smootheness compared to SS CaCO_3_, probably a result of different synthetic methods.

Indeed, atomic force microsope (AFM) demonstrated surface roughness of each CaCO_3_ (Figure 1) and showed remarkably smooth surfaces of SS CaCO_3_ compared with others. Calculated surface roughness parameters, *R*_a_ were 36.4, 7.9 and 1.8 nm for food bulk CaCO_3_, food nano CaCO_3_, and SS CaCO_3_, respectively. It has been reported that surface roughness of nanoparticles affected their biological behaviors including cytotoxicity [24], as their surface roughness minimize repulsive force between nanoparticles and plasma membrane, possibly influencing membrane damage or cellular uptake [25]. Thus, different surface roughness as well as particle size could result in different biological responses. It is worth noting that food grade CaCO_3_ particles were produced by the top-down of grinding sea shells and SS CaCO_3_ was obtained by the bottom-up of high gravity reactive precipitation. Zeta potentials of food bulk CaCO_3_, food nano CaCO_3_, and SS CaCO_3_ were −3.7 ± 1.9, 15.7 ± 0.5, and 11.8 ± 0.8 mV, respectively, indicating that surface charge of CaCO_3_ nanoparticles was different from bulk CaCO_3_. On the other hand, solubility of all CaCO_3_ particles was less than 0.01 and 0.1% (*w*/*v*) in physiological fluid at pH 7.0 and simulated gastric fluid at pH 1.5, respectively, suggesting that extremely low amount of CaCO_3_ particles can be partially dissolved into ions even under gastric conditions, regardless of particle size.

**Figure 2:**
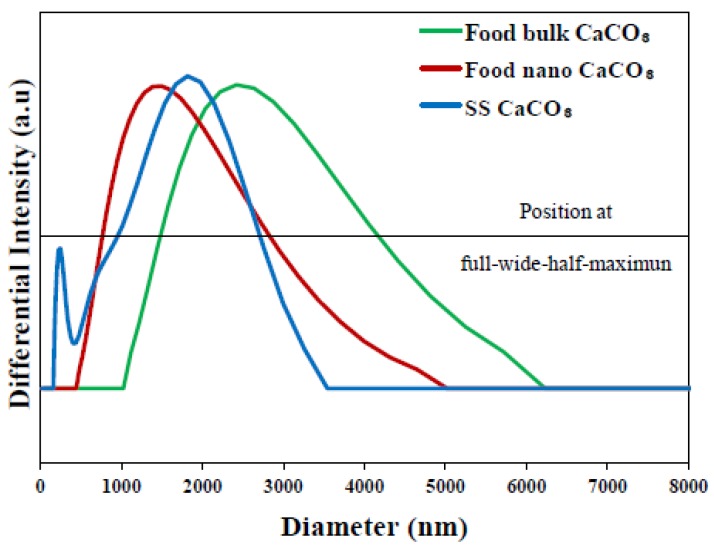
Hydrodynamic diameter of food bulk CaCO_3_ (dashed line), food nano CaCO_3_ (solid line) and SS CaCO_3_ (dotted line) as a function of differntial intensity. Horizontal line stands for the position of full-width at half-maximum to evaluate peak broadness.

In order to investigate hydrodynamic radii of CaCO_3_ particles after *in vivo* administration, we measured dynamic light scattering (DLS) pattern of CaCO_3_ particles in albumin solution (Figure 2). Although larger hydrodynamic diameters of all particles compared with their primary particle size (Figure 1) were observed, it was clearly noted that average value and distribution was different with respect to particle size and manufacturing method. Food bulk CaCO_3_ showed large diameter up to 6000 nm with wide full-width at half-maximum (FWHM), whereas those of food nano and SS CaCO_3_ were smaller and more narrow than bulk particles. SS CaCO_3_ particles showed slightly narrow FWHM value compared to food nano CaCO_3_, possibly due to the homogeneous particle size distribution as observed in SEM (Figure 1).

### 2.2. Cytotoxicity

#### 2.2.1. Cell Proliferation

**Figure 3:**
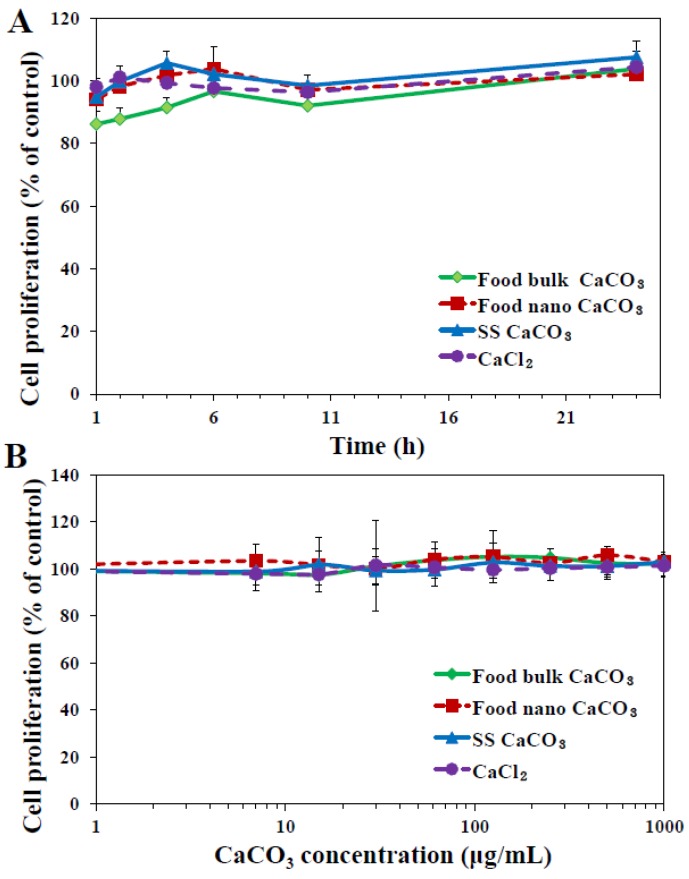
Effect of three different types of CaCO_3_ particles on cell proliferation of human intestinal INT-407 cells, as measured by water-soluble tetrazolium salts (WST-1) assay. (**A**) Cell proliferation exposed to 250 μg/mL particles or an equivalent amount of CaCl_2_ (based on calcium content) for 1–24 h; (**B**) Cell proliferation treated with different concentrations of CaCO_3_ particles or CaCl_2_ for 24 h.

To evaluate the effect of CaCO_3_ particles on cytotoxicity with respect to particle size, cell proliferation was measured with water-soluble tetrazolium salt (WST-1) assay in human intestinal INT-407 cells. In all experiments, an equivalent amount of CaCl_2_ as Ca^2+^ ions was used to allow cytotoxicity and uptake behaviors of CaCO_3_ particles and Ca^2+^ ions to be compared. As shown in Figure 3A, cell proliferation was not affected by all three different CaCO_3_ particles when the cells were exposed to 250 μg/mL particles for 1–24 h. Furthermore, no effect of particle size of CaCO_3_ on cell proliferation was found up to the highest concentration tested, 1000 μg/mL (Figure 3B), after 24 h of incubation, indicating their low cytotoxicity. Further cell exposure to nanoparticles for 72 h did not cause inhibition of cell proliferation (data not shown). Ca^2+^ ions did not exhibit cytotoxicity as well under the same experimental condition.

#### 2.2.2. Reactive oxygen species (ROS) Generation and lactate dehydrogenase (LDH) Release

**Figure 4:**
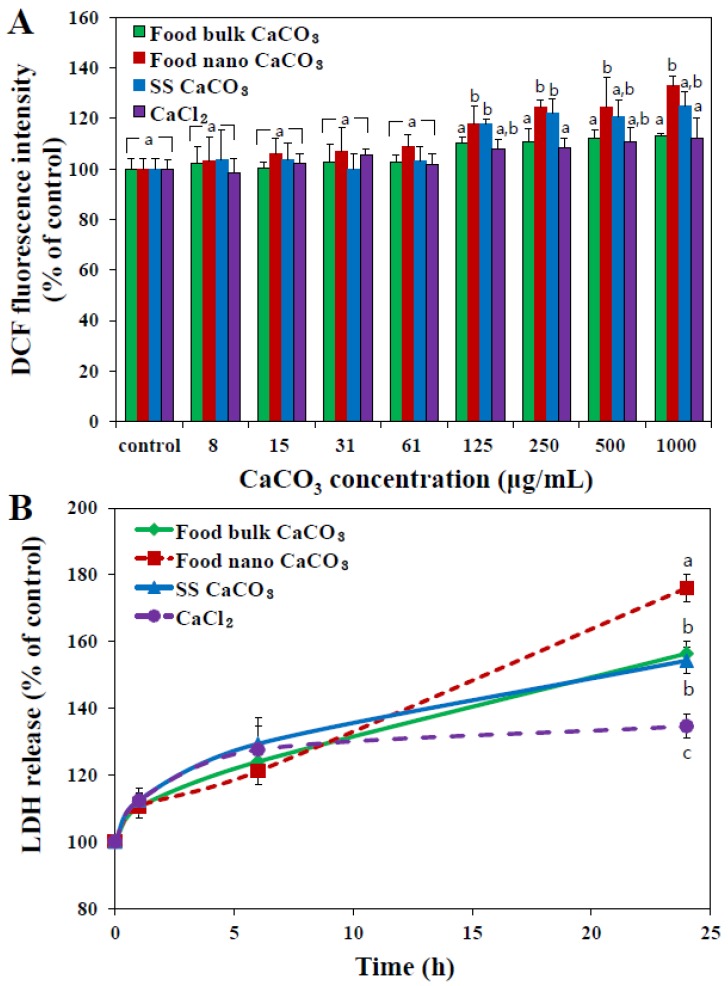
Effect of three different types of CaCO_3_ particles or an equivalent amount of CaCl_2_ (based on calcium content) on (**A**) ROS generation and (**B**) lactate dehydrogenase (LDH) release from human intestinal INT-407 cells after 24 h of incubation. The mean values with different letters (a, a,b, b, c) at the same concentration or time points indicate statistically significant difference (*p* < 0.05).

Generation of intracellular reactive oxygen species (ROS) was monitored using a cell permeant fluorescent probe. Figure 4A demonstrates that ROS significantly increased in INT-407 cells exposed to nano-sized materials, both food nano CaCO_3_ and SS CaCO_3_, at above 125 μg/mL. In particular, slightly high ROS generation was induced by food nano CaCO_3_ compared to SS CaCO_3_ at high concentration of 500–1000 μg/mL. Interestingly, food bulk CaCO_3_ nor Ca^2+^ ions did not generate ROS, suggesting that nanoparticles were more cytotoxic than bulk materials or Ca^2+^ ions.

When released levels of intracellular lactate dehydrogenase (LDH) into the extracellular medium was evaluated (Figure 4B), the highest LDH leakage was induced by food nano CaCO_3_, followed by food bulk CaCO_3_ ≈ SS CaCO_3_ > CaCl_2_. Taken together, food nano CaCO_3_ exhibited the highest cytotoxicity in terms of ROS generation and membrane damage, although it did not inhibit cell proliferation (Figure 3). It seems that CaCO_3_ nanoparticles can damage the cell membrane and consequently induce ROS, but these cytotoxic effects are not severe to affect cell prolliferation. It was reported that layered double hydroxide nanoparticles did not block cell proliferation up to 500 μg/mL, but caused ROS generation and LDH release [26,27]. Slightly high cytotoxicity of food nano CaCO_3_ is likely to be associated with surface roughenss resulting from the grinding process of sea shells as shown in AFM images and height profile in Figure 1B. Rough surface of food nano CaCO_3_ could maximize attractive interaction between particles and cellular membranes [25], subsequently inducing more oxidative stress as well as membrane damage than smooth surfaced SS CaCO_3_. Meanwhile, it should be noted that an equivalent amount of Ca^2+^ ions caused little cytotoxicity, and all CaCO_3_ particles had extremely low solubility. Therefore, cytotoxicity of CaCO_3_ particles seems to be related to their particulate fate under cell culture conditions.

### 2.3. Cellular Uptake Behaviors

#### 2.3.1. Cellular Uptake

**Figure 5:**
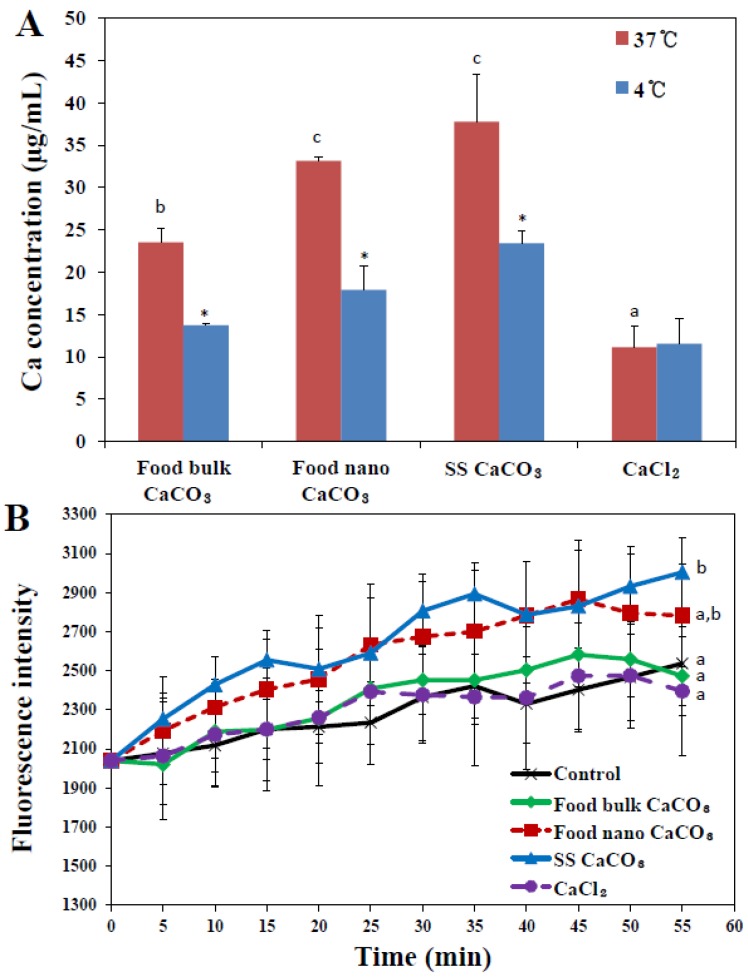
(**A**) Cellular internalization of three different types of CaCO_3_ particles or an equivalent amount of CaCl_2_ (based on calcium content) in human intestinal INT-407 cells after 2 h of incubation, as measured by inductively coupled plasma-atomic emission spectroscopy (ICP-AES); (**B**) intracellular Ca^2+^ levels monitored with Calcium Green™^−1^ probe (Life Technologies, Carsbad, CA, USA). The mean values with different letters (a, a,b, b) at the same temperature or time points indicate statistically significant difference (*p* < 0.05). * denotes significant difference in uptake amount between 37 and 4 °C (*p* < 0.05).

Cellular uptake of CaCO_3_ particles was evaluated by measuring total calcium levels in particle-treated INT-407 cells using inductively coupled plasma-atomic emission spectroscopy (ICP-AES), in order to investigate the effects of particle size on cellular internalization. Figure 5A shows that cellular internalization of CaCO_3_ particles remarkably increased as particle size decreased under nomal condition at 37 °C after 2 h of incubation, as evidenced by significantly high uptake of both food nano CaCO_3_ and SS CaCO_3_ compared to that of food bulk CaCO_3_. The cellular uptake behaviors showed correlation with specific surface area of CaCO_3_ particles, in other words, particles with larger surface area had higher cellular uptake. Furthermore, CaCO_3_ particles were more massively internalized into cells than Ca^2+^ ions, indicating different uptake pathways between particles and Ca^2+^ ions.

When the role of energy-dependent internalization in paricle uptake was examined by incubating the cells at 4 °C (Figure 5A), cellular uptake of all CaCO_3_ particles significantly decreased in comparison with that obtained at 37 °C, regardless of particle size, showing 41.65%, 45.96%, and 37.93% inhibitions for food bulk CaCO_3_, food nano CaCO_3_, and SS CaCO_3_, respectively. This result suggests that all CaCO_3_ particles can partially enter the cells by energy-dependent endocytosis. Uptake of Ca^2+^ ions was not affected by low temperature, probably attributed to their different internalization pathway from that for CaCO_3_ particles, which does not need energy for uptake.

On the other hand, when intracellular uptake of CaCO_3_ particles was monitored with the Ca^2+^ probe (Figure 5B), significantly elevated Ca^2+^ levels were found inside the cells treated with SS CaCO_3_ and food nano CaCO_3_. Since the Ca^2+^ probe detects only ionized Ca^2+^ ions from CaCO_3_ particles, thus elevated intracellular Ca^2+^ levels in the presence of nanoparticles suggest that SS CaCO_3_ and food nano CaCO_3_ can be more effectively taken up by cells and easily dissolved into Ca^2+^ ions inside the cells than food bulk CaCO_3_. Meanwhile, a significant difference in Ca^2+^ levels between CaCO_3_ nanoparticles and Ca^2+^ ions is in good agreement with cellular uptake measured by ICP-AES (Figure 4A), which can be explained by efficient cellular uptake behaviors of CaCO_3_ nanoparticles as compared with Ca^2+^ ions.

#### 2.3.2. Intestinal Transport

**Figure 6:**
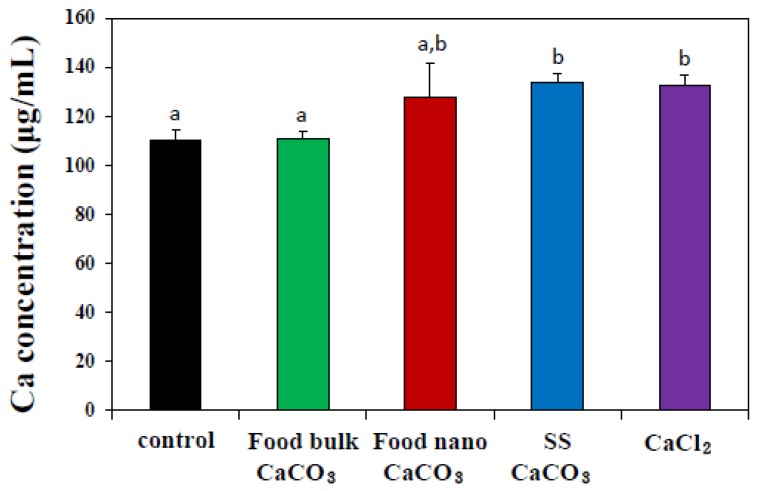
Intestinal transport of three different types of CaCO_3_ particles or an equivalent amount of CaCl_2_ (based on calcium content) by microfold (M) cells using an *in vitro* model of human FAE after 6 h of incubation, as measured by inductively coupled plasma-atomic emission spectroscopy (ICP-AES). The mean values with different letters (a, a,b, b) in tested groups indicate statistically significant difference (*p* < 0.05).

Further mechanistic study on the transport of three different CaCO_3_ particles across the intestinal epithelium was carried out using 3D cell culture system, *in vitro* model of human intestinal follicle-associated epithelium (FAE), based on co-culture of human intestinal epithelial Caco-2 cells and human Raji B lymphocytes [28,29]. The FAE is different from normal intestinal epithelium and contains specialized microfold (M) cells that are capable of transporting a wide range of materials, such as bacteria, viruses, macromolecules, and particles [30,31]. Thus, the role of M cells in *in vivo* particle absorption across the intestinal epithelium can be evaluated using the *in vitro* FAE model. Figure 6 shows that the transport of food nano CaCO_3_, SS CaCO_3_, and CaCl_2_ by M cells significantly increased, while elevated transport of food bulk CaCO_3_ was not found, suggesting that M cells are the transport mechanism for both CaCO_3_ nanoparticles and Ca^2+^ ions. In particular, the transport of SS CaCO_3_ was similar to that of CaCl_2_. Hence, it seems that reagent grade SS CaCO_3_ with a smooth surface and narrow size distribution compared to food nano CaCO_3_ (Figure 1) is more favorable to be transported by M cells.

On the other hand, this result also implies that bulk particles cannot be transcytosed by M cells, possibly leading to low *in vivo* oral absorption efficiency. It is worth noting that the same tendency was obtained for food bulk CaCO_3_ in Figure 5B and Figure 6, showing neither elevated intracellular Ca^2+^ levels nor increased intestinal transport, whereas, significant cellular uptake was measured by ICP-AES analysis (Figure 5A). It is probable that bulk materials are somewhat adsorbed on the cell plasma membrane, which may result in totally elevated false cellular uptake by ICP-AES, although 5 mM EDTA was treated to remove particles not taken up by the cells. Here, Figure 5B only measured intracellular Ca^2+^ levels, while Figure 6 represented total transported calcium amount into basolateral solution in FAE model, reflecting intestinal absorption by M cells. Little cellular uptake, but high intestinal transport of Ca^2+^ ions, as shown in Figure 5 and Figure 6, implies that extremely low levels Ca^2+^ ions are taken up by cells in ionic state, probably due to calcium homeostasis, but they can be efficiently transported through the intestinal epithelium.

### 2.4. Biokinetics

**Figure 7:**
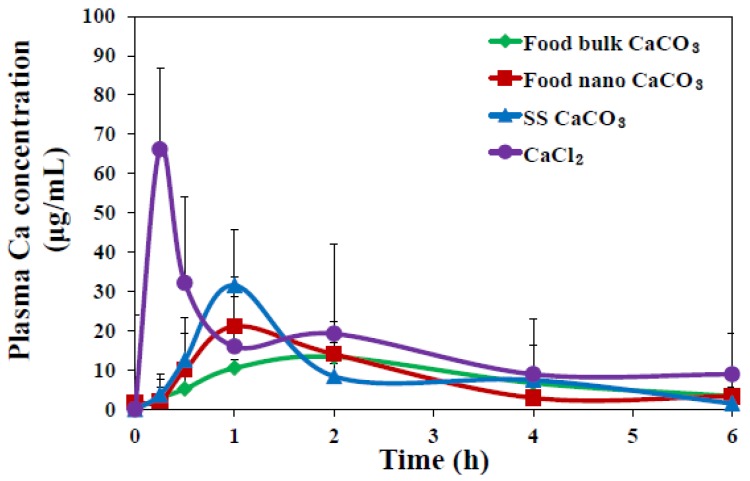
Plasma concentration-time curves of three different types of CaCO_3_ particles (250 μg/mL) or an equivalent amount of CaCl_2_ (based on calcium content) after a single-dose oral administration to female rats. Biokinetic data are presented as increase in calcium levels after subtracting the basal plasma calcium levels detected in untreated controls.

*In vivo* oral absorption of CaCO_3_ particles was also evaluated following a single-dose oral administration to rats. Figure 7 demonstrates different plasma concentration-time curves of three CaCO_3_ particles; SS CaCO_3_ and food nano CaCO_3_ particles showed more rapid absorption, showing peak concentration at 1 h *versus* 2 h for food bulk CaCO_3_. Interestingly, slightly high peak concentration at 1 h was found for SS CaCO_3_ than food nano CaCO_3_ and retarded decrease in peak concentration was observed for food nano CaCO_3_ compared to SS CaCO_3_. This might be explained by the different hydrodynamic size distribution in spite of similar specific surface area values between two nanoparticles, as shown in Figure 2; SS CaCO_3_ with narrow size distribution might be absorbed faster than food nano CaCO_3_, while food nano CaCO_3_ having larger hydrodynamic size is absorbed more slowly. The delayed absorption profile was also found for food bulk CaCO_3_, showing *T*_max_ value at 2 h (Figure 7). On the other hand, Ca^2+^ ions were determined to behave differently from CaCO_3_ particles, with the highest maximum concentration at 15 min.

When biokinetic parameters of CaCO_3_ particles were compared (Table 1), significantly increased *C*_max_ and shortened *T*_1/2_ and MRT values were examined for SS CaCO_3_ compared to food nano CaCO_3_ and food bulk CaCO_3_. Nevertheless, total oral absorption was not affected by particle size or surface roughness, as shown in similar AUC values and about 5% oral absorption for all CaCO_3_ particles. It is strongly likely that nanoparticles can more rapidly enter the systemic circulation than bulk-sized materials; however, particle size of CaCO_3_ does not influence total oral absorption efficiency. On the other hand, remarkably high oral absorption of Ca^2+^ ions as compared with CaCO_3_ particles was found, indicating different biological fates between CaCO_3_ particles and Ca^2+^ ions.

**Table 1:** Biokinetic parameters and oral absorption of calcium carbonate (CaCO_3_) particles after oral administration to rats.

Biokinetic parameters	Food bulk CaCO_3_	Food nano CaCO_3_	SS CaCO_3_	CaCl_2_
*C*_max_ (μg/mL)	13.39 ± 1.63 ^c^	21.55 ± 6.71 ^b,c^	31.56 ± 0.99 ^b^	66.16 ± 12.98 ^a^
*T*_max_ (h)	2.00 ^c^	1.00 ^b^	1.00 ^b^	0.25 ^a^
AUC (h × μg/mL)	63.21 ± 2.04 ^b^	62.26 ± 2.08 ^b^	66.40 ± 8.60 ^b^	120.98 ± 11.14 ^a^
*T*_1/2_ (h)	2.50 ± 0.01 ^c^	2.86 ± 0.22 ^d^	1.59 ± 0.01 ^b^	0.97 ± 0.07 ^a^
MRT (h)	4.58 ± 0.18 ^c^	4.28 ± 0.23 ^b^	2.94 ± 0.07 ^a^	2.81 ± 0.20 ^a^
Absorption (%) ^1^	4.86 ± 0.16 ^b^	4.79 ± 0.16 ^b^	5.11 ± 0.66 ^b^	8.07 ± 0.74 ^a^

Notes: ^1^ Absorption (%) was calculated based on AUC values. The mean values with different letters (^a^, ^b^, ^c^) in each column are significantly different at *p* < 0.05.

## 3. Experimental Section

### 3.1. Materials and Characterization

The bulk- and nano-sized food grade CaCO_3_ materials (food bulk CaCO_3_, food nano CaCO_3_) produced by grinding sea shells were purchased from Apexel Co., Ltd. (Pohang, Korea). Reagent grade CaCO_3_ (SS CaCO_3_), which was produced by high gravity reactive precipitation [32], was puarchased from Skyspring Nanomaterials Inc. (Houston, TX, USA). Particle size and morphology were examined by SEM (Hitachi S-4300, Tokyo, Japan). Surface roughness of each sample (0.1 g/mL), dispersed in ethanol, was measured with AFM (NX10, Park Systems, Suwon, Korea) and a drop of suspension was located on a flat silicon wafer. The surface charge (zeta potential) and size distribution of the particles were determined using a zeta potentiometer (Zetasizer Nano ZS system, Malvern Instruments, Worcestershire, UK). Specific surface areas were determined using N_2_ adsorption-desorption isotherms using a micromeritics ASAP 2020 (Accelerated Surface Area and Porosimetry System, Micromeritics Instrument Corporation, USA). Surface area values of CaCO_3_ were calculated with Brunauer-Emmett-Teller (BET) method. Adsorption isotherms below relative pressure 0.14 (for bulk CaCO_3_) and 0.25 (for food nano and SS CaCO_3_) were utilized for BET plot. In order to evaluate hydrodynamic size distribution of CaCO_3_ in physiological condition, each sample (1000 μg/mL) was dispersed in bovine serum albumin solution (200 μg/mL). Hydrodynamic size measurement was performed with DLS apparatus ELS-Z100 (Otsuka Electronics Co., Ltd., Osaka, Japan) three times with a refractive index of water 1.330.

Solubility was measured by dispersing 5 mg/mL particles in phosphate buffered saline (PBS, pH 7.0) or simulated gastric fluid (0.034 M sodium chloride, 3.2 g pepsin, pH 1.5). After incubation for 6 h at 37 °C, supernatants were collected by ultracentrifugation (15,000× *g* at 15 min). Then, calcium concentrations in the supernatants were determined by ICP-AES (JY2000 Ultrace; HORIBA Jobin Yvon, Stow, MA, USA).

### 3.2. Cell Culture

Human intestinal epithelial (INT-407) cells were provided by Dr. Tae-Sung Kim at Korea University (South Korea) and cultured in MEM (Welgene Inc., Daegu, Korea) under a humidified atmosphere (5% CO_2_/95% air) at 37 °C. The medium was supplemented with 10% heat inactivated fetal bovine serum (Welgene Inc., Daegu, Korea), 100 units/mL penicillin, and 100 μg/mL streptomycin.

### 3.3. Cell Proliferation

Effect of particles on cell proliferation was measured using the WST-1 assay (Roche, Basel, Switzerland). Briefly, cells (5 × 10^3^/100 μL) were exposed to 1–1000 μg/mL particles for 24 h or to 250 μg/mL for times ranging from 1 to 24 h. An equivalent amount of CaCl_2_ solution (based on calcium content) was also prepared for comparison. Next, 10 μL of WST-1 solution (Roche) was added to each well, and cells were further incubated for 4 h. Absorbance was then measured using a plate reader at 440 nm (Dynex Technologies, Chantilly, VA, USA). Cells incubated in the absence of particles were used as the control. The experiment was repeated three times on three separate days.

### 3.4. Intracellular ROS Generation

Intracellular ROS levels were monitored using a peroxide-sensitive fluorescent probe, carboxy-2ʹ,7ʹ-dichlorofluorescein diacetate (H_2_DCFDA, Molecular Probes, Eugene, OR, USA), according to the manufacturer’s guidelines. Briefly, cells (5 × 10^3^/100 μL) were incubated with the particles or an equivalent amount of CaCl_2_ (based on calcium content) for 24 h, washed with PBS, collected by centrifugation, and incubated with 40 μM carboxy-H2DCFDA for 60 min at 37 °C. After washing with PBS, dichlorofluorescein fluorescence was immediately measured using a fluorescence microplate reader (SpectraMax^®^ M3, Molecular Devices, Silicon Valley, CA, USA), and excitation and emission wavelengths were 490 and 530 nm, respectively. Cells not treated with particles were used as the control. The experiment was repeated three times on three separate days.

### 3.5. LDH Leakage

The release of LDH was monitored with the CytoTox 96 Non-Radioactive Cytotoxicity assay (Promega, Madison, WI, USA). Cells (5 × 10^4^ cells/1 mL) were incubated with 250 μg/mL CaCO_3_ materials or an equivalent amount of CaCl_2_ (based on calcium content) for times ranging from 1 h to 24 h. Then, the plates were centrifuged, and aliquots (50 μL) of cell culture medium were collected from each well and placed in new microtiter plates. Then, 50 μL of substrate solution was added to each well and the plates were further incubated for 30 min at room temperature. Finally, after adding the 50 μL of stop solution, the absorbance at 490 nm was measured with a microplate reader (SpectraMax^®^ M3, Molecular Devices, Silicon Valley, CA, USA). Cytotoxicity is expressed relative to the basal LDH release from untreated control cells. The experiment was repeated three times on three separate days.

### 3.6. Cellular Uptake

Cells (1 × 10^6^/mL) were incubated overnight under the standard condition as described above, then replaced with fresh medium containing 250 μg/mL CaCO_3_ materials or an equivalent amount of CaCl_2_ (based on calcium content) for 2 h. Cells were then washed three times with PBS and treated with 5 mM EDTA for 40 s to remove particles not taken up by the cells. Higher EDTA concentration for more prolonged time was found to cause membrane damage. After washing three times with PBS, cells were harvested by scraping and centrifuged. The cell pellets thus obtained were digested in 3 mL of ultrapure nitric acid, treated with 0.5 mL of H_2_O_2_, and heated at about 160 °C. Each mixture was heated until the samples were completely digested. The remaining solution was then removed by heating until the solutions were colorless and clear. The solution were finally diluted to 5 mL with D.D.W. and filtered with 0.45 μm. Calcium concentrations were determined by ICP-AES (JY2000 Ultrace; HORIBA Jobin Yvon, Stow, MA, USA). Cells incubated in the absence of particles were used as controls.

In order to determine the role of energy-dependent endocytosis in CaCO_3_ uptake, the uptake experiment was also performed at 4 °C and CaCO_3_ uptake was analyzed by ICP-AES in the same manner. On the other hand, intracellular Ca^2+^ levels resulted from uptake of CaCO_3_ materials or an equivalent amount of CaCl_2_ (based on calcium content) were monitored with Calcium Green™^−1^ probe (Life Technologies, Carsbad, CA, USA). Cells (5 × 10^4^ cells/1 mL) were incubated with 250 μg/mL CaCO_3_ materials or an equivalent amount of CaCl_2_ (based on calcium content) for 60 min in the presence of 10 μM probe. The fluorescence was immediately measured using a fluorescence microplate reader (SpectraMax^®^ M3, Molecular Devices, Silicon Valley, CA, USA), and excitation and emission wavelengths were 506 nm and 531 nm, respectively. Cells not treated with particles were used as the control. All experiments were repeated three times on three separate days.

### 3.7. Intestinal Transport Mechanism

For mechanistic study on intestinal transport, an *in vitro* model of human intestinal FAE was prepared according to the protocol developed by des Rieux *et al.* [28,29]. Human intestinal epithelial Caco-2 cells were purchased from the Korean Cell Line Bank (Seoul, Korea) and grown in DMED supplemented with 10% fetal bovine serum, 1% non-essential amino acids, 1% L-glutamine, 100 units/mL penicillin, and 100 μg/mL streptomycin under the standard condition as described above. Briefly, Transwell^®^ polycarbonate inserts (Corning Costar, New York, NY, USA) were coated with Matrigel™ basement membrane matrix (Becton Dicknson, Bedford, MA, USA), prepared in pure DMEM, and then placed at room temperature for 1 h. Supernatants were removed and inserts were washed with DMEM. Caco-2 cells (5 × 10^5^ cells) were grown on the upper insert side and incubated for 14 days. Then, non-adherent human Burkitt’s lymphoma Raji B cells (5 × 10^5^ cells, Korean Cell Line Bank, Seoul, Korea) in the same medium were added to the basolateral insert compartment, and the co-cultures were maintained for 5 days. CaCO_3_ materials (250 μg/mL) or an equivalent amount of CaCl_2_ (based on calcium content) were prepared in Hank’s balanced salt solution buffer, and apical medium of the cell monolayers were replaced by a particle suspension and incubated for 6 h. Basolateral solutions were then sampled and the concentration of transported particles were estimated by measuring total calcium levels with ICP-AES as described above. The experiment was repeated three times on three separate days.

### 3.8. Oral Absorption

Six female rats per group were administered a single dose of 250 mg/kg of the three CaCO_3_ or an equivalent amount CaCl_2_ by oral gavage; controls (*n* = 6) received an equivalent volume of 0.9% saline. All animal experiments were performed after obtaining approval from the Animal and Ethics Review Committee of Seoul Women’s University. Body weight changes, behaviors, and symptoms were carefully recorded daily after treatment. To determine plasma calcium concentrations, blood samples were collected via a tail vein at 0, 0.25, 0.5, 1, 2, 4, and 6 h of post-oral administration. Blood samples were centrifuged at 3000× *g* for 15 min at 4 °C to obtain plasma. The following pharmacokinetic parameters were estimated using Kinetica version 4.4 (Thermo Fisher Scientific, Waltham, MA, USA): maximum concentration (*C*_max_), time to maximum concentration (*T*_max_), area under the plasma concentration-time curve (AUC), half-life (*T*_1/2_), and mean residence time (MRT). The plasma samples were quantitatively analyzed by ICP-AES as described above.

### 3.9. Statistical Analysis

The statistical analysis was performed using the Student’s *t*-test for unpaired data, and one-way analysis of variance (Tukey’s test, version 11.0) was conducted using SAS software (SAS Institute, Cary, NC, USA) to determine the significance of differences between experimental groups. All results are presented as the mean ± standard deviation and *p* < 0.05 were considered to be statistically significant.

## 4. Conclusions

We investigated the effect of particle size (bulk *versus* nano) of food grade CaCO_3_, produced by grinding sea shells, on cytotoxicity, cellular uptake, intestinal transport, and *in vivo* oral absorption. A comparative study with bottom-up synthesized reagent grade SS CaCO_3_ nanoparticles or Ca^2+^ ions was also performed. The physicochemical characterization revealed that food nano CaCO_3_ was well produced with an average size of ~100 nm, but it had morphological rough surface compared to SS CaCO_3_. Both food nano CaCO_3_ and SS CaCO_3_ nanoparticles exhibited slightly high toxicity compared to food bulk CaCO_3_ in terms of ROS generation and LDH release. Cellular uptake behaviors of CaCO_3_ particles were different from Ca^2+^ ions, showing significantly increased uptake and energy-dependent endocytic pathways, especially for nano-sized particles. The M cells were determined to be an important intestinal transport mechanism of CaCO_3_ nanoparticles, in a similar manner as Ca^2+^ ions did, implying that CaCO_3_ nanoparticles can be efficiently transcytosed across the intestinal epithelium. *In vivo* biokinetic study demonstrated more rapid absorption of CaCO_3_ nanoparticles than food bulk CaCO_3_, but total absorption efficiency was not affected by particle size. Biokinetics of CaCO_3_ particles were different from Ca^2+^ ions, suggesting that the biological fate of CaCO_3_ particles was primarily a particulate form, regardless of particle size, showing a slower absorption rate and lower total oral absorption efficacy than Ca^2+^ ions. These results will be useful to understand and predict potential toxicity and oral efficacy of food grade nanoparticles.
